# New York Society Reports

**Published:** 1887-03

**Authors:** 


NEW YORK SOCIETY REPORTS.

In this number will be found the report of the special meeting of the First District Dental Society of New York. Our right to publish these reports has, we understand, been questioned. It is well known that a bargain has been made with the S. S. White Dental Manufacturing Co., which gives to *The Cosmos* the official reports of this society, and that of the Odontological Society of New York. We have no desire to trench upon the rights of others, and certainly would not print what we have if we thought we had no privilege in the matter. This journal is not published by men who have no sense of honor, or who would engage in defrauding a respected contemporary, nor is it reduced to such extremities that these or any other particular reports are necessary to its existence.

As we understand it, these New York societies hold open meetings. All are invited to attend. If one of the New York daily papers desired to make a report of the proceedings, its representative would be welcomed, and why should the privilege be denied to a respectable journal of their own profession? These societies do not profess to hold their sessions for the exclusive benefit of any one person or journal. Some of the publishers of this journal are active in their support, and hold official positions in them. They fully comprehend that all the official papers, the report which is made by the properly appointed officer for *The Cosmos,* and all the regular and voluntary addresses which may be made, are the property of that journal.

But does this agreement forbid another journal to make an abstract of the proceedings at its own expense, and to publish such syllabus, all the time fully respecting the exclusive right of *The Cosmos* to publish the papers in full and to have sole access to the official records ? If it be so, we shall certainly abide by the terms of that agreement, for we wish only that to which we are honestly entitled. Some of the publishers of this journal have read papers before the New York societies, but their manuscript was never permitted to be seen by the editor, nor were they even asked to make an abstract for him. Usually, very little reference was made to such papers, lest it be thought that an unfair advantage was taken. If any abstract was given it was brief, and obtained precisely as the other matter was—at our own expense.

Dental societies are supposed to exist for the furtherance of professional ends, and for the spread of scientific information. If the scope of any society contemplates a selfish object, it should be so stated in its laws, that dentists who contribute papers may have a fair understanding of the matter. If a society proposes to sell its knowledge, or to make merchandise in any way of the free contributions of its members and others, it should be so nominated in the bond. If a specialist in any department makes a long journey, and is at considerable expense to demonstrate some new principle in dental science, he usually does so because in that way he can best communicate the intelligence to the world. His address is intended for the profession at large, and not for any clique or segregated part of it. He does not anticipate that the society will close up any avenues of information, but that they will allow the widest publicity, for his labors are for the public good.

Again, the members of dental societies are supposed to be laboring, not for their own selfish benefit, but for the good of all—to advance the interests of the profession at large. Any other theory would imply a retrogradation to the dark days when the doors of every dentist were closed against his brother, and were only open to the sesame of money; when knowledge was bought and sold, and when there was no such thing as true professional feeling. Our present greatest boast is, that among no class of men is there more of the true spirit of professional ethics, and it is to this fact that we owe our wonderful advance. Every progressive dentist comprehends that his status is only to be permanently raised by elevating his whole profession. We believe, then, that the true spirit of dentistry is to give the widest possible publicity to all professional knowledge.

But every society has the undoubted right to select an official medium for its official communications, and that right should be respected by all. The American Dental Association has exercised this right, but it has never been understood that the bargain with the S. S. White Dental Manufacturing Co. precluded other journals from publishing a fair abstract of the proceedings, nor has their right to do this been questioned. When it is, we shall say that the society has no longer an excuse for existence, for it will be violating all the true ethics which it is supposed that it is its mission to maintain. We have always thought that the publishers of its transactions had the hardest end of the bargain, and believed that they made it more to help the society than for any other reason, and we have always questioned the propriety of accepting that aid. The society is not supposed to be a money-making concern, and when the principle which controls the publication of its transactions is penuriousness or avariciousness on its part, it is pursuing an unworthy course.

We have written this in a spirit of frankness and honesty, and let it not be understood that we are in the remotest degree implying any censure of the S. S. White Dental Manufacturing Co. They have been entirely honorable and generous, so far as we know, and have never made any complaint to us whatever. But we would not have them, or any one else, think that what we do has been done in a clandestine manner, or with the desire or intent of inflicting an injury upon a respected and revered contemporary. There has never been any ungenerous rivalry between *The Cosmos* and this journal, and we trust there never will be. There is room for both, and we hope that the regard we have for that great journal and its editor is, in a measure at least, a mutual feeling. The only complaint that we have heard concerning the publication of the reports of the New York societies comes from those who, we think, do not quite comprehend the matter, and it is for the purpose of pointing out the stand-point from which we view it that this article has been written.

